# Effect of Stroke on Fall Rate, Location and Predictors: A Prospective Comparison of Older Adults with and without Stroke

**DOI:** 10.1371/journal.pone.0019431

**Published:** 2011-04-29

**Authors:** Lisa A. Simpson, William C. Miller, Janice J. Eng

**Affiliations:** 1 Graduate Program in Rehabilitation Sciences, University of British Columbia, Vancouver, Canada; 2 Department of Occupational Science and Occupational Therapy, University of British Columbia, Vancouver, Canada; 3 Department of Physical Therapy, University of British Columbia, Vancouver, Canada; Virginia Commonwealth University Rehabilitation and Research Center, United States of America

## Abstract

**Background:**

The literature suggests that stroke is a major risk factor for falls, but there is a lack of prospective, controlled studies which quantify fall-risk after stroke. The purpose of this study was to compare the rates, location and predictors among individuals recently discharged home from stroke rehabilitation to age and sex matched controls.

**Methodology/Principal Findings:**

A sample of 80 people with stroke and 90 controls received baseline assessments of balance, mobility and balance confidence. Falls were recorded prospectively over 13 months for both groups. Group differences in fall rates and contribution of clinical measures to falls were determined using negative binomial regression. Fall location was compared between groups using χ^2^ statistics. The rate of falls for individuals with stroke was 1.77 times the rate for the control group. People with stroke were more likely to fall at home. Poorer balance (Berg Balance Scale) was associated with greater falls for both stroke and control groups (incidence rate ratio [IRR]: 0.908 and IRR: 0.877 respectively). A faster Timed Up and Go Test was associated with greater falls for the stroke group (IRR: 0.955) while better walking endurance (Six Minute Walk Test) was associated with greater falls for the controls (IRR: 1.004). Balance confidence was not an independent predictor in either group.

**Conclusions:**

Individuals recently discharged home are at greater risk of falling than individuals without stroke. Attention to home environment is warranted. Balance function can predict falls for both people with stroke and age and sex matched controls. Increased mobility may increase exposure to fall opportunities.

## Introduction

Following a stroke, people are at high risk of falls (1.3–6.5 falls/person/year) [1], with the highest rates occurring upon discharge from hospital (8.7 falls/person/year) [2]. Falls may lead to fractures [3], fear of falling [4], activity restriction [5] and depression [2]. Creating improved fall prevention strategies early after stroke requires an accurate understanding of fall risks and predictors.

No prospective studies have compared the rate or location of falls between stroke survivors recently discharged home and matched controls. Thus, it is not known whether these individuals fall at a higher rate than the general older population who fall at a rate of 0.3–1.6 falls/person·year [6]. Importantly, no study following patients early after stroke has accounted for the geographical [7] and temporal variance [8] of fall rates with the inclusion of a control group. In fact, there has been only one controlled, prospective study assessing fall risk by Jorgenson et al. [9] and they found that stroke survivors with chronic stroke (mean 10 years post-stroke) fell 3.57 times more than an age and gender matched control group.

Moreover, there is a lack of evidence concerning the unique effects of modifiable risk factors such as balance, mobility and balance confidence on the frequency of falls after recent stroke. Prospective studies that have examined the contribution of balance and/or mobility to falls in this population of stroke survivors have conflicting results and do not determine their unique contributions by including both mobility and balance in their predictor models [2], [10], [11]. Also, no prospective study to date has examined the contribution of balance confidence to falls among people with recent stroke. Greater understanding of the relative contribution of modifiable risk factors to falls after stroke can lead to the development and study of fall prevention interventions for this population. Indeed, a 2010 meta-analysis of fall prevention intervention studies highlights the shortage of high quality studies that examine multifactorial interventions to prevent falls after stroke [12].

Thus, the purpose of this prospective study is to compare: (1) rates of falls (2) location of falls and (3) contribution of balance, mobility and balance confidence to falls between individuals recently discharged from stroke rehabilitation and age and gender matched controls.

## Methods

### Design

This project utilized a longitudinal inception cohort design that followed a group of individuals for one year after a first stroke from the time of discharge to the community. We also followed an age and sex matched control group of individuals who had not experienced a stroke for one year. The study was approved by the University of British Columbia Clinical Research Ethics Board and by the ethics committees for Vancouver Coastal Health, Interior Health and Vancouver Island Health authorities.

### Participants

A total of 98 individuals with stroke and 110 control individuals were recruited between September 2004 and July 2008. Potential participants with stroke were recruited through inpatient rehabilitation units in five hospitals. The inclusion criteria were: 1) discharged from a rehabilitation facility to their own home in the community, 2) had a history of a single stroke as identified by a neurologist with CT or MRI, 3) were able to walk for a minimum of 10 meters with or without assistive devices, 4) age ≥50 years 5) were able to communicate in English and provide informed written consent.

Population controls who reported not having a stroke were recruited through advertisements in local newspapers and community centers. The controls were then frequency-matched to the stroke group by gender and age. Individuals from either group were excluded if they had significant musculoskeletal or neurological conditions other than stroke and lived >50 km from the data collection centers. All participants gave informed written consent prior to participating in the study.

### Clinical assessments

Baseline clinical assessments were performed within 4 weeks upon discharge from rehabilitation for the stroke group and upon recruitment for the controls. Assessments were conducted by trained research assistants for both groups. Participant characteristics (age, sex and cognition) and the variables of interest (balance, mobility and balance confidence) were assessed at baseline. Cognition was assessed using the Cognitive Capacity Screening Examination (CCSE) [13]. Balance was assessed using the Berg Balance Scale (BBS) [14]. The BBS consists of 14 tasks (eg. reaching, turning and balancing on one leg), resulting in a maximum score of 56 indicating better balance. Mobility was assessed using the Timed Up and Go Test (TUG) [15] and the Six Minute Walk Test (6MWT) [16]. The TUG was used to measure functional mobility and represents the time it takes in seconds for a subject to stand up from a chair, walk 3 meters, turn around, walk back to the chair and sit down. The 6MWT was used to measure walking endurance and it represents the distance in meters that subjects can walk in 6 minutes. Balance confidence was measured using the Activities-Specific Balance Confidence Scale (ABC) [17]. The ABC is a self-report questionnaire that asks subjects to rate their confidence (from 0 to 100%) in performing 16 functional activities without losing their balance. Higher scores indicate higher balance confidence.

### Falls

Falls were defined as unintentionally coming to rest on the ground, floor or lower level that is not a result of a seizure, stroke/myocardial infarction or a major displacing force (e.g. earthquake) [18]. Monthly fall diaries, considered the gold standard of fall measurement [19] were used to capture fall incidence. The circumstances and consequences related to each fall event were also recorded. Each month, participants who failed to send in a fall calendar were followed up with a phone call. At baseline, participants with stroke reported how many times they fell during the interim time between discharge from rehabilitation and the baseline appointment (average length was 4.4 weeks). Individuals in the control group reported how many times they fell during the last month at baseline. Participants in both cohorts (stroke, control) were followed up for 12 months from baseline, therefore supplying 13 fall diaries.

### Data Analysis

Descriptive statistics, t-tests or Mann-Whitney U tests, and chi-square statistics were used to analyze baseline characteristics of both groups. Chi-square statistics were used to determine differences in fall location between the stroke and control group. First, negative binomial regression was used to compare differences in fall rates between the cohorts (stroke, control) when controlling for sex and age. Second, the sample was then divided based on population (stroke, control), and two separate multiple negative binomial regressions were performed to determine the predictors of falls for each cohort. The initial models input the variables of interest (balance, mobility, balance confidence) while controlling for age, sex and cognition. The final models were obtained by using the variables that minimized Akaike's Information Criterion (AIC) [20]. The resulting parameter coefficients obtained in the final models were translated into the estimated number of falls associated with the variable of interest when the other variables were held constant at their means [20]. Negative binomial regression has been identified as the appropriate method for analyzing recurrent events such as falls [21], [22]. Moreover, recent literature examining statistical methods for analyzing falls has underlined the flaws associated with categorical analyses (e.g., faller versus non-faller or repeated faller versus single faller) [21]. While negative binomial regression allows for the inclusion of subjects with varied follow up times [22], we excluded subjects with greater than 25% missing falls data or subjects with less than 6 months of fall diaries. Finally, outliers were removed based on their undue influence on the regression results. Data was analyzed using SPSS 18.0 and Stata 11.0 and an alpha of 0.05 was used for all analyses.

## Results

**Figure 1:**
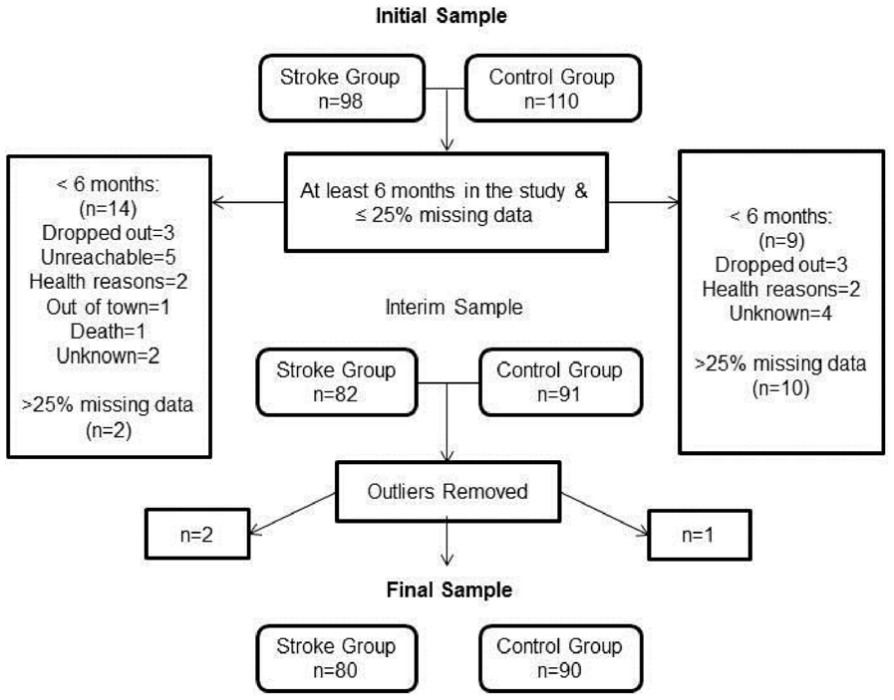
Flow diagram of final sample used in data analysis.

A total of 80 individuals with stroke and 90 controls were included in the analysis which represented 82% of the original sample. There were no statistical differences between the included and excluded sample (Table S1). Twelve months was the average follow up time for the final sample. The patient flow diagram is outlined in Figure 1. Fall and injury incidence along with the fall circumstances are outlined in Table 1. Individuals with stroke experienced a total of 109 falls while the control group experienced a total of 70 falls. The individuals in the stroke group had significantly lower balance, mobility and balance confidence than the control group (Table 2). In addition, baseline balance, mobility and balance confidence scores among many individuals with stroke fell below reported clinical thresholds with 28 (35%) people below the 45 threshold score for the BBS [14] indicating increased fall risk, 40 (50%) individuals above the 15 seconds for the TUG indicating increased fall risk [23] and 62 (78%) below the 400 meter threshold for the 6MWT indicating increased risk for mortality [24]. The 6MWT values of the control group were similar to reference values provided for a similar age group [25] and were approximately double that of the stroke group. Also 23 (29%) people in the stroke group obtained ABC scores below 50 indicating low levels of physical functioning [26].

**Table 1:** Fall incidence, circumstances and injuries by fall status for both cohorts.

	Stroke			Control		
	(n = 80)			(n = 90)		
	No fall	One fall	Multiple Falls	No fall	One fall	Multiple falls
	n(%)			n(%)		
**Fall incidence**	40 (50)	14 (18)	26 (32)	52 (58)	22 (24)	16 (18)
**Fall incidence w/injury**		4 (29)	15 (58)		9 (41)	11 (69)
**Injuries**		4 (29)	24 (25)		9 (41)	17(35)
fractures		1	3		0	2
bruising		2	12		5	5
strain/sprain		–	2		1	5
cuts/scrapes		–	3		3	4
dislocation		–	–		–	1
unspecified		1	4		–	–
**total # of injuries**		28 (26)		26 (37)		
**Fall circumstances**		14 (13)	95 (87)		22 (31)	48 (69)
**location**						
at home		9 (64)	55 (58)		6 (28)	16 (33)
indoors		2 (14)	13 (14)		8 (36)	2 (4)
outdoors		3 (22)	23 (24)		8 (36)	28 (58)
not specified			4 (4)			2 (4)
**activity**						
walking		4	22		10	23
standing		3	12		3	2
turning		3	17		1	5
transferring		2	15		2	1
bending over		1	10		–	2
stairs		1	5		1	8
Other* †			7		4	5
unknown			7		1	2
**total # of falls**		109			70	

*Other, Stroke group: squatting (5); stepping on chair/step ladder (2).

† Other, Control group: running (4); hiking (2); pushups (1); yoga (1); carrying a ladder (1).

**Table 2:** Baseline characteristics and demographics for both cohorts.

	Stroke	Control	
	(n = 80)	(n = 90)	p-value*
**Mean Age (SD)**	67.6 (9.9)	68.4 (10.0)	0.70
**Gender:Female (%)**	22 (27.5)	31 (34.4)	0.33
**Mean CCSE score(SD)**	26.0 (3.1)	28.1 (1.7)	<0.001
**Mean BBS score(SD)**	46.1 (8.3)	54.3 (3.2)	<0.001
**Mean TUG (SD)**	20.0 (14.3)	8.2 (1.8)	<0.001
**Mean 6MWT (SD)**	275.9 (141.8)	527.8 (85.9)	<0.001
**Mean ABC score(SD)**	62.7 (24.2)	93.2 (10.9)	<0.001

CCSE: Cognitive Capacity Screening Examination (0–30); BBS: Berg Balance Scale (0–56); TUG: Timed Up and Go (in seconds); 6MWT: Six Minute Walk Test (in metres); ABC: Activity-Specific Balance Confidence Scale (0–100).

*p-values determined using a t-test for normally distributed data, Mann-Whitney U Test for non-normally distributed data or chi-square test for proportions.

### Fall Rates and location

Individuals with stroke fell at a rate of 1.77 times that of the control group during the study period (Table 3A). Sex and age were not significantly associated with falls. Examination of the location of the falls revealed that the individuals who experienced a stroke fell more at home than the control group with 59% of the falls experienced by the stroke group occurring at home compared to 31% of the falls by the control group (χ^2^ = 12.71, p<0.001) (Table 1).

**Table 3:** Final multiple negative binomial regression models for predicting falls.

Variable	β	IRR	95% Confidence Interval	p-value
A: Stroke as predictor of falls (n = 170, combined stroke and control groups)				
**Stroke**	0.569	1.767	1.149–2.716	0.009
**Gender: Male**	0.271	1.312	0.813–2.116	0.266
**Age**	0.018	1.018	0.995–1.041	0.121

B: Predictors of falls among individuals with a history of stroke (n = 80)				
**Age**	0.025	1.025	0.993–1.058	0.124
**CCSE**	0.091	1.095	0.985–1.217	0.093
**BBS**	−0.096	0.908	0.845–0.976	0.009
**TUG**	−0.047	0.955	0.914–0.997	0.038

C: Predictors of falls among controls (n = 90)				
**BBS**	−0.131	0.877	0.793–0.970	0.011
**6MWT**	0.004	1.004	1.000–1.009	0.034

β: regression coefficient; IRR: incidence rate ratio; CCSE: Cognitive Capacity Screening Examination; BBS: Berg Balance Scale; TUG: Timed-Up and Go Test; 6MWT: Six Minute Walk Test.

### Fall Predictors

For the stroke group, the BBS and TUG were significant predictors, while age and cognition remained in the final model, but did not reach statistical significance (Table 3B). For the control group, the BBS and 6MWT were the only two significant predictors of falls in the final model (Table 3C).

**Figure 2:**
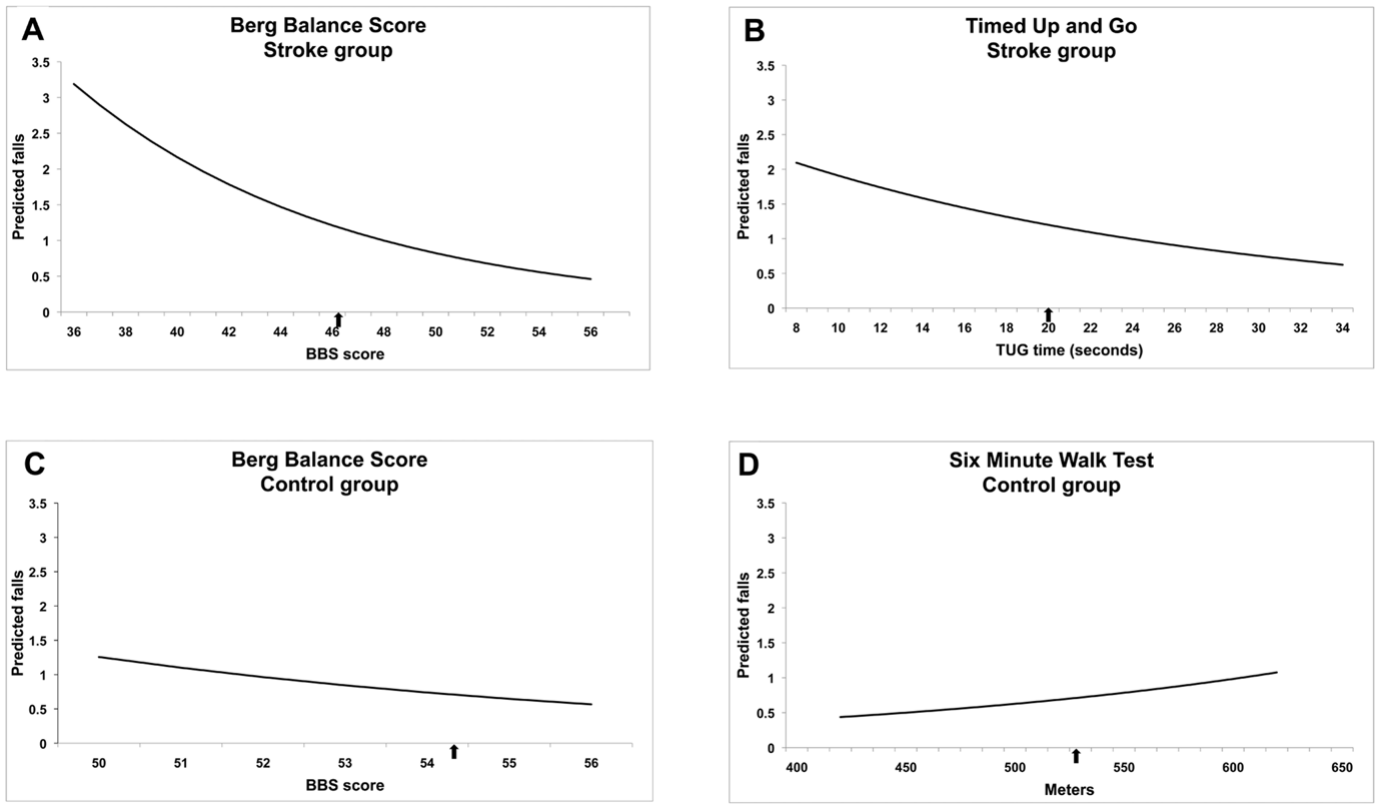
Estimated relationship between clinical measures and number of falls. Estimated relationship between falls and independent clinical measure predictors for the stroke group and the control group. Each plot displays predicted number of falls at different levels of the clinical measure scores while holding all other variables in the model at their mean. Estimates are plotted for scores falling between the 10^th^ and 90^th^ percentile of the sample scores. The arrow on the x-axis indicates the sample mean.

The BBS was thus the only common predictor between the control and stroke groups with better balance resulting in fewer falls (Table 3). Despite having an 8 point difference in mean Berg scores between the stroke and control group, the BBS score associated with more than one fall was similar between the two groups. While holding the other variables included in the model at their means [20], a BBS score below 52 was associated with more than 1 fall for the controls, while a BBS score below 49 was associated with more than 1 fall for the stroke group (Figure 2A,C). However, the predicted falls increased sharply for the stroke group as the BBS score fell below 44.

Unexpectedly, a decrease in TUG was associated with an increase in falls (Table 3B) for the stroke group, and an increase in 6MWT was associated with an increase in falls for the control group (Table 3C). In both cases, the effect size was small as the IRRs were significant but close to 1.0. In the stroke group, a decrease in TUG from 34 to 8 seconds (10^th^ to 90^th^ percentile), when balance, cognition and age were held at their means, resulted in a predicted increase of approximately 1.5 falls (Figure 2B). In the control group, increasing the 6MWT from 425 meters to 625 meters (10^th^ to 90^th^ percentile), when balance is held at its mean, resulted in a predicted increase of approximately 0.7 falls (Figure 2D).

## Discussion

Our results demonstrate that recent stroke is an important risk factor for falls. We found that individuals with stroke fell 1.77 times more than the age and gender matched controls over the period of 13 months. Rates from three prospective, but not controlled studies investigating people with recent stroke range from 1.29 to 5.0 falls/person·year [2], [5], [27]. The average rate of 1.4 falls/person·year found in our sample of individuals with stroke lies within the lower end of this range. Our results accurately document a higher risk of falls among this population and is the first prospective study to examine the fall rates among people recently discharged home from stroke rehabilitation when controlling for falls among the older adult population in the same geographical range and over the same period of time.

Our results suggest that people recently discharged from stroke rehabilitation to home are at greater risk for falls in their home. Following a stroke, people might be more likely to spend more time at home or be more cautious when outside of their home. Clinically, this finding underlies the importance of home assessment, home safety education and environmental modifications as part of discharge planning.

Balance was the only common independent predictor of falls in both individuals with and without a history of stroke. Our findings suggest that the difference between fall rates between the groups can be explained by the difference in balance scores.

The association between mobility and falls in prospective studies examining fall predictors among community-dwelling individuals with stroke has been tenuous [2], [10], [28], [29]. Only one study found a relationship between mobility and falls when controlling for other clinical variables [28]. However, mobility was defined dichotomously as ability/non ability to rise from a chair. This aspect of mobility makes up only one portion of the TUG, which was used to conceptualize mobility in our study. A 2011 study by Persson et al. [29] found that an increase in TUG time was associated with an increased risk of falls after recent stroke. The study sample included individuals who were non-ambulatory however (29% of the sample). This is in contrast to our study in which every individual was able to ambulate independently. We found the TUG to be an independent predictor of falls among people with recent stroke. The direction of its effect was positive however, and thus opposite to what was expected. A possible explanation for this observation is that increased mobility (as reflected by the TUG), once already ambulatory, is associated with increased exposure to fall opportunities [30], [31].

This is the first known study to examine balance confidence as a predictor of falls post-stroke. An association between balance confidence and falls after stroke has been found in recent cross-sectional studies [32], [33]. The direction of the association however cannot be determined using a cross-sectional study design. Balance confidence was not an independent predictor of falls among our sample of individuals with recent stroke. Given the complexity of falling among individuals with a stroke history, it is possible that balance confidence does not have a direct relationship with falls but mediates an effect through the interaction with other variables. For instance, given a particular level of mobility, balance confidence could help to explain the types of activities a person with recent stroke engages in which could in turn influence one's risk of falling.

In summary, our study found that people recently discharged home from stroke rehabilitation fall at a greater rate, and more at home than the general older adult population. Intervention studies among this population should therefore consider falls as an important follow up outcome. Our results can only be generalized to ambulatory individuals recently discharged home from stroke rehabilitation. However, as the majority of people who survive a stroke eventually regain the ability to ambulate independently [34], our results are relevant to a large proportion of stroke survivors. Our study was able to detect unique effects of mobility and balance on falls for individuals with stroke and controls as described in our results. Given a larger sample size however, we might have attained increased power to detect smaller unique effects of the other clinical variables on falls. Finally, a 2010 meta-analysis on interventions trials to reduce falls after stroke has called for consistent and accurate methods of reporting and analyzing falls [12]. Findings from studies that explore predictors of falls help guide fall prevention trials and thus should adhere to these standards. Therefore, given the complexity of fall risk prediction after recent stroke, further investigation using prospective designs with appropriate methods of analysis is warranted.

## Supporting Information

Table S1 Baseline characteristics of included and excluded participants for both cohorts.
(DOC)
