# Supplementary material for: Effect of Stroke on Fall Rate, Location and Predictors: A Prospective Comparison of Older Adults with and without Stroke
Source: PLoS One. 2011 Apr 29;6(4):e19431. doi: 10.1371/journal.pone.0019431 (PMC3084849; doi:10.1371/journal.pone.0019431)
Supplement: Table S1 — Baseline characteristics of included and excluded participants for both cohorts. (DOC) [file pone.0019431.s001.doc]

Table S1: Baseline characteristics of included and excluded participants for both cohorts.

|  | **Stroke** | | | **Control** | | |
| --- | --- | --- | --- | --- | --- | --- |
|  | **Included** | **Excluded** |  | **Included** | **Excluded** |  |
|  | **(n=80)** | **(n=18)** | **p-value** | **(n=90)** | **(n=18)** | **p-value** |
| **Mean Age (SD)** | 67.6 (9.9) | 66.7 (11.3) | 0.73 | 68.2 (10.0) | 67.4 (9.5) | 0.75 |
| **Gender: Female (%)** | 22 (27.5) | 5 (27.8) | 0.98 | 31 (34.4) | 4 (22.2) | 0.31 |
| **Mean CCSE score (SD)** | 26.0 (3.1) | 23.5 (7.1) | 0.44 | 28.1 (1.7) | 27.7 (2.8) | 0.88 |
| **Mean BBS Score (SD)** | 46.1 (8.3) | 41.6 (10.7) | 0.06 | 54.3 (3.2) | 54.9 (1.8) | 0.35 |
| **Mean TUG (SD)** | 20.0 (14.3) | 27.4 (28.8) | 0.18 | 8.2 (1.8) | 8.3 (2.1) | 0.88 |
| **Mean 6MWT (SD)** | 275.9 (141.8) | 230.8 (126.0) | 0.24 | 527.8 (85.9) | 524.7 (109.4) | 0.81 |
| **Mean ABC Score (SD)** | 62.7 (24.2) | 58.2 (22.9) | 0.43 | 93.2 (10.9) | 94.1 (5.5) | 0.54 |

CCSE: Cognitive Capacity Screening Examination (0-30); BBS: Berg Balance Scale (0-56); TUG: Timed Up and Go (in seconds); 6MWT: Six Minute Walk Test (in metres); ABC: Activity-Specific Balance Confidence Scale (0-100)

* p-values determined using a t-test for normally distributed data, Mann-Whitney U Test for non-normally distributed data or chi-square test for proportions.
